# Digital Phenotyping for Differential Diagnosis of Major Depressive Episode: Narrative Review

**DOI:** 10.2196/37225

**Published:** 2023-01-23

**Authors:** Eric Ettore, Philipp Müller, Jonas Hinze, Matthias Riemenschneider, Michel Benoit, Bruno Giordana, Danilo Postin, Rene Hurlemann, Amandine Lecomte, Michel Musiol, Hali Lindsay, Philippe Robert, Alexandra König

**Affiliations:** 1 Department of Psychiatry and Memory Clinic University Hospital of Nice Nice France; 2 Research Department Cognitive Assistants Deutsches Forschungszentrum für Künstliche Intelligenz GmbH Saarbrücken Germany; 3 Department of Psychiatry and Psychotherapy Saarland University Medical Center Hombourg Germany; 4 Department of Psychiatry Hopital Pasteur University Hospital of Nice Nice France; 5 Department of Psychiatry School of Medicine and Health Sciences Carl von Ossietzky University of Oldenburg Bad Zwischenahn Germany; 6 Research Department Sémagramme Team Institut national de recherche en informatique et en automatique Nancy France; 7 Research Department, Cognition-Behaviour-Technology Lab University Côte d’Azur Nice France; 8 Research Department Stars Team Institut national de recherche en informatique et en automatique Sophia Antipolis - Valbonne France

**Keywords:** depression, bipolar disorder, posttraumatic stress disorder, differential diagnosis, digital phenotyping, speech analysis, nonverbal behavior, physiological measures, posttraumatic stress disorder, mental health, clinical interview, diagnosis, mental disorder, interview, digital health, psychotrauma, digital, information

## Abstract

**Background:**

Major depressive episode (MDE) is a common clinical syndrome. It can be found in different pathologies such as major depressive disorder (MDD), bipolar disorder (BD), posttraumatic stress disorder (PTSD), or even occur in the context of psychological trauma. However, only 1 syndrome is described in international classifications (Diagnostic and Statistical Manual of Mental Disorders, Fifth Edition [DSM-5]/International Classification of Diseases 11th Revision [ICD-11]), which do not take into account the underlying pathology at the origin of the MDE. Clinical interviews are currently the best source of information to obtain the etiological diagnosis of MDE. Nevertheless, it does not allow an early diagnosis and there are no objective measures of extracted clinical information. To remedy this, the use of digital tools and their correlation with clinical symptomatology could be useful.

**Objective:**

We aimed to review the current application of digital tools for MDE diagnosis while highlighting shortcomings for further research. In addition, our work was focused on digital devices easy to use during clinical interview and mental health issues where depression is common.

**Methods:**

We conducted a narrative review of the use of digital tools during clinical interviews for MDE by searching papers published in PubMed/MEDLINE, Web of Science, and Google Scholar databases since February 2010. The search was conducted from June to September 2021. Potentially relevant papers were then compared against a checklist for relevance and reviewed independently for inclusion, with focus on 4 allocated topics of (1) automated voice analysis, behavior analysis by (2) video and physiological measures, (3) heart rate variability (HRV), and (4) electrodermal activity (EDA). For this purpose, we were interested in 4 frequently found clinical conditions in which MDE can occur: (1) MDD, (2) BD, (3) PTSD, and (4) psychological trauma.

**Results:**

A total of 74 relevant papers on the subject were qualitatively analyzed and the information was synthesized. Thus, a digital phenotype of MDE seems to emerge consisting of modifications in speech features (namely, temporal, prosodic, spectral, source, and formants) and in speech content, modifications in nonverbal behavior (head, hand, body and eyes movement, facial expressivity, and gaze), and a decrease in physiological measurements (HRV and EDA). We not only found similarities but also differences when MDE occurs in MDD, BD, PTSD, or psychological trauma. However, comparative studies were rare in BD or PTSD conditions, which does not allow us to identify clear and distinct digital phenotypes.

**Conclusions:**

Our search identified markers from several modalities that hold promise for helping with a more objective diagnosis of MDE. To validate their potential, further longitudinal and prospective studies are needed.

## Introduction

Major depression is a frequent syndrome affecting more than 264 million people worldwide [1]. Major depressive episode (MDE), as defined by the current criteria [2], describes a large heterogeneous clinical syndrome comprising more than 1490 combinations of symptoms [3]. The possibility of fulfilling MDE criteria with opposite symptoms, such as insomnia and hypersomnia, decrease/increase in appetite or agitation, and psychomotor retardation, highlights this heterogeneity. This heterogeneity could explain why studies with large samples of patients with major depression show that only 30% of them remitted with a first-line antidepressant and another 30% did not remit after 4 consecutive treatment trials [4].

These various “profiles” of MDE may require different therapeutic approaches. In fact, according to international classifications [2], MDE symptoms are the same whether in major depressive disorder (MDD) or in bipolar disorder (BD). In clinical practice, hypomanic or manic episodes have been the most recognizable characteristics of BD, while depression seems to be most frequent clinical manifestation [5,6]. Performing differential diagnosis between MDD and BD during an MDE is challenging. Approximately 20% of people with MDE would be misdiagnosed as MDD when the correct diagnosis is BD [7]. Thereby, the delay for a correct BD diagnosis can vary from almost 7 to 10 years after the first mood symptoms [8]. These misdiagnoses can have several damaging consequences, for example, induction of manic, hypomanic, or mixed states; development of treatment resistance; or cycle acceleration [9].

Furthermore, Rytwinski et al [10] showed in their meta-analysis that almost 52% of patients with posttraumatic stress disorder (PTSD) have comorbidity with MDD. In fact, in the Diagnostic and Statistical Manual of Mental Disorders, Fifth Edition (DSM-5), several symptoms are shared between MDD and PTSD [11] and a study by Gros et al [12] showed that in patients classified by DSM-5 as having MDD and PTSD, only the presence of trauma (criterion A of DSM-5) could differentiate MDD from PTSD efficiently. Moreover, patients classified as comorbid between MDD and PTSD showed the highest severity of symptoms, more cognitive deterioration, higher suicidality, and worse prognosis compared with noncomorbid patients [12]. One possible explanation could be the time-dependent fluctuation of PTSD symptoms [13]. In a dynamic model, MDD could be a progression or an evolution of psychotraumatism [14]. Although there is a frequent association between MDD and PTSD, the link between them remains unclear. However, it points to the importance of exploring the presence of psychological trauma and PTSD in patients with MDE because it carries the potential to drastically impact further therapeutic care. Misdiagnosing PTSD or the presence of psychological trauma could lead to a worse prognosis due to inappropriate drug treatment or psychotherapy.

In this article, we focus on the use of new digital markers in MDE, given their potential to serve as an additional and objective diagnostic support. For this, we explore studies in MDE, especially when it is presented in the context of either MDD or BD, as well as when it is presented alongside the presence of psychological trauma or PTSD.

Digital phenotyping refers to the moment-to-moment quantification of human behavior in everyday life using data from digital devices [15]. It suggests the possibility of revealing clinically relevant information by a continuous and nonintrusive monitoring of behavioral and mental states. Two categories of data can be captured by digital phenotyping. First, active data, which require the input of the participants being studied. It includes, for example, recordings, responses to surveys, and social media activity. Passive data, however, do not require the individual’s participation to be captured. They include, for example, accelerometer-derived data or GPS coordinates [16]. Digital markers correspond to disease indicators obtained digitally, which can be used to define a digital phenotype [17]. It refers to the possibility of capturing, through computerized measurement tools, certain symptoms or behavior specific to a psychiatric disorder.

There is a specific interest in psychiatry in which symptoms and clinical states are mainly measured using question-based scales and without biological markers. Thus, the interest of digital phenotyping would be to obtain objective and quantifiable measurements of these symptoms or behaviors [16]. Moreover, diagnostic categories do not capture the heterogeneity of symptoms, and variability between patients can lead to misdiagnosis and incorrect treatments [18]. Identifying objective markers of clinical states, including trans-diagnostic symptoms, could improve disease classification and treatment [19].

Thus, digital phenotyping should play a role in routine clinical practice, especially by improving clinical diagnosis and treatment by an early detection of condition onset, by assessing treatment response, or even by detecting relapse [20]. Therefore, we assume that new digital measures may provide indicators for the heterogeneous characteristics of MDE and could help to better distinguish between its potential different clinical profiles. Based on this rationale, we performed a narrative review including studies on several technologies, such as speech and video analysis and physiological measures, namely, heart rate variability (HRV) and electrodermal activity (EDA) for the assessment of MDE.

## Methods

### Search Strategy

We conducted this narrative review from June to September 2021, which mainly concerned the current application of digital tools for MDE diagnosis. The following electronic databases were searched: PubMed/MEDLINE, Web of Science, and Google Scholar. The review was limited to articles in English or French and because we aimed to establish an overview of the most recent advances in these domains, we restricted our search to studies published after January 2010. For this paper, we decided to concentrate on digital tools that are easy to use during a clinical interview, minimally invasive, and least dependent on compliance. Thus, we focused on 4 types of digital markers: (1) automated voice analysis, behavior analysis by (2) video and physiological measures, (3) HRV, and (4) EDA. We then used broad search terms to capture as many studies as possible that are specifically related to these technologies and we associated them with terms related to specific psychiatric issue. We concentrated on MDE and on several psychiatric conditions where it may occur, especially on (1) MDD, (2) BD, (3) PTSD, and (4) psychological trauma. The search strings are included in Multimedia Appendix 1.

### Inclusion and Exclusion Criteria

The inclusion criteria were as follows: primary articles or reviews dealing with depression, BD, bipolar depression, PTSD, or psychological trauma using digital tools for speech or video analysis or analysis of physiological parameters such as HRV or skin conductance. The exclusion criteria were as follows: articles related to activity monitoring on social networks or by SMS text messages or phone and articles related to ecological momentary assessment. There is an abundance of literature on these topics, but we wanted to focus here on digital tools that can be used specifically during a social interaction such as an interview with the clinician. Unrelated and redundant articles or studies using technologies for therapeutic purposes were also excluded. Theoretical papers, study protocols, letters, books or book chapters, statistical reviews, and dissertations were also excluded.

### Study Selection

Three authors (EE, AK, and PR) independently screened relevant titles and abstracts for this narrative review. Then, all 3 authors screened relevant papers for eligibility. Finally, according to inclusion and exclusion criteria, full texts of eligible articles were obtained (EE).

## Results

### Overview

We selected 74 articles dealing with the use of the 4 aforesaid digital tools for diagnosis assessment under different psychiatric conditions. A total of 39 articles were selected for MDD, 18 for BD, and 17 for PTSD and psychological trauma. Results are summarized on 3 tables. See Table 1 for MDD, and Multimedia Appendix 2 for BD, PTSD and psychological trauma.

**Table 1:** Summary of speech analysis, nonverbal behavioral analysis, heart rate variability (HRV), and electrodermal activity studies (EDA) in patients with major depressive disorder (MDD).

Study			Participants		Recording setting		Principal findings		Principal features
**Speech analysis studies**									
	Alghowinem et al [21]	30 with MDD and 30 in HC^a^		Reading tasks with negative and positive meaning Free speech by answering questions, where the patients describe events that had aroused significant emotions		MFCC^b^, jitter, shimmer, energy, and loudness features were robust in getting the general characteristic of depressive speech		Prosodic, spectral, and source features	
	Arevian et al [22]	15 with MDD, 14 with BD^c^, 14 with schizophrenia, and 14 with affective schizophrenia		Free speech (answering questions from an app with phone call)		Correlation with providers’ global assessment: ↓ Negative emotional language ↑ Positive emotional language ↑ Complex word use		Type of words used	
	Hönig et al [23]	219 participants with BDI (BD type I) assessment		Read speech, spontaneous speech, telling an imagined story		Depression severity: ↓ Average and SD MFCC 2 ↓ SD MFCC 3 ↓ Average pitch ↓ Shimmer ↓ Spectral harmonicity ↓ Speech rate (↑ Average syllables duration)		Prosodic, spectral, source, and filter features	
	Horwitz et al [24]	35 participants with Hamilton Rating Scale for Depression and Quick Inventory of Depressive Symptomatology assessment		Free speech, reading, and distinct vowels		Depression severity: ↑ Jitter and shimmer ↓ Harmonic-to-noise ratio ↓ First and third formants ↓ Speech rate ↓ (Read) and ⇧ (free speech) pitch-based features		Prosodic, spectral, source, and filter features	
	Mundt et al [25]	105 with MDD		Free and with tasks: reciting the alphabet, counting, reading, and sustained vowels		Correlation with depression severity: ↑ Total speech time ↑ Total pause time ↑ Variable pause length ↑ Percentage pause time ↓ Speech pause ratio ↓ Speaking rate		Temporal and prosodic features	
	Quatieri et al [26]	35 with MDD		Free speech for prosodic measurements and distinct vowels for shimmer, jitter, and aspiration measurements		Depression severity and psychomotor retardation: ↑ Shimmer ↑ Aspiration ↓ Harmonic-to-noise ratio Depression severity ↑ Jitter ↓ Pitch variance ↓ Average velocity		Prosodic, source, and filter features	
	Shinohara et al [27]	30 with MDD and 14 in HC		Free speech and reading phrases aloud Detection of emotional elements (ie, “anger” and “joy”) 2 indices extracted: “vitality” and “mental activity”		MDD correlated with: ↓ Vitality scores (effect size 1.03 and AUC^d^ 0.76, sensitivity 0.93, and specificity 0.55)		Temporal and “emotional” prosodic features	
	Taguchi et al [28]	36 with MDD and 36 in HC		3 tasks: reading task, verbal fluency task, and reading task again		Significant discrimination between MDD and HC with MFCC 2 (sensitivity 77.8% and specificity 86.1%)		Spectral features	
	Wang et al [29]	47 with MDD and 57 in HC		4 tasks: video watching, question answering (natural speech and interview), text reading, and picture describing Each task involved 3 emotional materials: positive, negative, and neutral		Patients with MDD (no matter which emotion or task was involved, with a large effect size): ↓ Loudness ↓ MFCC 5 ↓ MFCC 7 ↓ Fundamental frequency (F0) and MFCC 3 (in some scenarios with moderate effect size)		Prosodic and spectral features	
	Xu et al [30]	45 with MDD, 43 with schizophrenia, and 41 in HC		Semistructured face-to-face interviews		Patients with MDD: ↑ % of words related to the past and sadness ↑ Conversational interruption ↑ Response time		Temporal and prosodic features, as well as the type of words used	
	Yamamoto et al [31]	97 with MDD, 68 with BD, and 76 in HC		10 minutes of free speech		Depression severity: ↓ Speech rate ↑ Pause time ↑ Response time		Temporal features	
**Nonverbal behavioral analysis studies**									
	Alghowinem et al [32]	Database with depression status (Quick Inventory of Depressive Symptomatology, 16 Items)		Free interview		Eye activity Head pose		Average recalls (mean of sensitivity and specificity) was 70% for detecting depression	
	Bhatia et al [33]	47 with MDD		No task Symptom severity was evaluated at 1, 7, 13, and 21 weeks		Head movement		Head movement synchrony did not change over the course of treatment with a change in depression severity	
	Dibeklioglu et al [34]	49 with MDD		No task Symptom severity was evaluated at 1, 7, 13, and 21 weeks		Facial movement Head movement Vocal prosody		For depression recognition: AUC was 67.25% for the fusion of head movement dynamics and vocal prosody AUC was 73.16% for facial movement dynamics and vocal prosody AUC was 77.77% for a combination of facial and head movement dynamics AUC was 78.67% by fusion of all modalities	
	Fiquer et al [35]	40 with MDD		Evaluation at baseline (T0) and after a 2 weeks’ tDCS^e^ treatment (T1) Describing their current mood during a 15-minute interview Face and trunk recording		Ethogram (21 questions), 20 nonverbal categories: 10 indicative of high-energy and favorable disposition to social interaction (eye contact, illustrative gestures, symmetric smile, raised eyebrows, yes/no nodding, head up, head to side, verbal backchannel, body posture toward the interlocutor); 10 indicative of low energy, negative feelings, or social disinterest (folded arms, head down, shrug, asymmetric smile, adaptive gestures, crying, frown, tight lips, lips down, silence); 1 verbal category: speaking		Clinical improvement: ↓ Head down, lips down, frown, and crying ↑ Yes nodding and eye contact Facial, head, and hand expressive movements were associated with the severity of depression	
	Fiquer et al [36]	78 patients with MDD: 50 from the Netherlands and 28 from Brazil		During the HDRS^f^ interview, face and trunk recording of both the patient and interviewer; scoring by a blinded observator		Speaking effort: patients’ behaviors Encouragement: interviewers’ behaviors		No association between behavioral variables and baseline severity of depression Patients who did not respond to treatment or did not remit: ⇧ speaking effort from before to after treatment	
	Fiquer et al [37]	100 with MDD and 83 in HC 41 with MDD treated by sertraline (25-200 mg) at the first hospital 59 with MDD; 23 treated by escitalopram (10-20 mg) and 36 treated by tDCS in a second hospital		Semistructured 15-minute interview with general questions, face and trunk recording 2 assessments: before treatment (T0) and after 8 weeks of treatment (T1) Scoring by a blinded observator		Ethogram (21 questions) from Fiquer et al [35]		Patients with MDD: ↑ Shrug, head, and lips down; adaptive hand gestures; frown, head, and lips down and cry ↓ Asymmetric smile, eye contact, and smile NVB^g^ was not associated with depression severity and did not significantly change after depression treatment Treatment responders at baseline: ↑ Interpersonal proximity; head down; adaptive hand gestures; frown, cry, and folded arms; head to side; no nodding ↓ Eye contact	
	Girard et al [38]	18 with MDD		Recording during the first 3 interview questions (depressed mood, feelings of guilt, and suicidal ideation)		Facial expressivity defined by the Facial Action Coding System in terms of individual muscle movements called AUs^h^		With symptom severity: ↓ AU 12 activity (smile and signal affiliative intent) ↑ AU 14 activity (contempt, negative affect, and signal nonaffiliative intent) ↑ Facial expressions associated with contempt	
	Girard et al [39]	33 with MDD		Recording during the first 3 interview questions (depressed mood, feelings of guilt, and suicidal ideation)		Facial expressivity defined by the Facial Action Coding System in terms of individual muscle movements called AUs		With symptom severity ↓ Affiliative facial expressions (AUs 12 and 15) ↑ Nonaffiliative facial expressions (AU 14) ↓ Head motion (ie, amplitude and velocity)	
	Guo et al [40]	52 males and 52 females in the depressed group (Patient Health Questionnaire–9 >5) 52 males and 52 females in HC (BDI <5)		Stimuli tasks of 3 emotional valences (watching film clips, replying to 9 free-response questions, reading 3 phonetically balanced passages containing affective content, and describing pictures)		Facial expressivity Audio		For depression recognition: Watching film clips showed the highest recognition rates (AUC up to 0.798 and 0.807) Positive emotional stimuli greater than negative emotional stimuli	
	Jiang et al [41]	12 with MDD Evaluated before and after deep brain stimulation		Free interview		Facial expressivity (7 basic emotions)		AUC 0.75 detecting response to treatment	
	Kacem et al [42]	49 with MDD		No task Symptom severity was evaluated at 1, 7, 13, and 21 weeks		Facial movement Head movement		For depression recognition: Facial movement was greater than head movement (AUC 66.19% vs 61.43%) With 2 modalities combined, AUC 70.83%	
**HRV studies**									
	Adolph et al [43]	85 outpatients with suicidal ideation		ECG^i^: At rest quietly for 3 minutes: “Resting HRV” After watching a sad film: “HRV reactivity”		HF^j^-HRV reactivity but not HF-HRV at rest was predictive of higher scores on suicidal ideation		Frequency domain: HF	
	Fernandes et al [44]	50 with MDD		ECG at rest Video for positive and negative NVB		HF and RMSSD^k^ were positively correlated with positive NVB Negative NVB was not associated with HRV		Frequency domain: HF and LF^l^ Time domain: RMSSD Ethogram (Fiquer et al [35])	
	Giurgi-Oncu et al [45]	78 with MDD		24-hour Holter ECG Evaluation at 1 and 6 months after therapy (sertraline)		↑ HRV at 1 and 6 months after selective serotonin reuptake inhibitors		Frequency domain: HF Time domain: SDNN^m^ and RMSSD	
	Hartmann et al [46]	62 with MDD and 65 in HC		2 time measures: before and after 2 weeks of antidepressant treatment 15 minutes of resting ECG 1-2 days before treatment		At baseline compared with HC: ↓ HRV (HF, LF, SD1^n^, and RMSSD) After treatment: HRV normalized in MDD for HF, LF, SD2^o^, and SD1/SD2 ratio		Frequency domain: LF, HF, and LF-to-HF ratio Time domain: SDNN, RMSSD, pNN50^p^ Nonlinear: SD1, SD2, SD1-to-SD2 ratio	
	Kircanski et al [47]	722 with MDD, 309 with anxious depression (according to the HDRS interview), and 413 without Outcomes at 8 weeks		2-minute seated ECG recording, first with eyes open and second with eyes closed		In anxious depression, better treatment response if: ↑ HRV in pretreatment In nonanxious depression, better treatment response if: ↓ HRV in pretreatment		Time domain: RMSSD Heart rate	
	Lee et al [48]	34 with MDD Outcome at 12 weeks of antidepressant treatment		5-minute ECG		Positive correlation at baseline: HDRS items 14 and 15 with LF-to-HF ratio Positive correlation at the endpoint: HDRS item 5 and LF HDRS items 7 and 13 (fatigue-related item) and LF HDRS item 8 and LF, SDNN, and RMSSD HDRS total, LF, and HF		Frequency domain: VLF^q^, LF, HF, LF-to-HF ratio Time domain: SDNN, RMSSD	
	Neyer et al [49]	50 with MDD		ECG at rest for the HRV measure Measures before and after treatment		Depressive symptoms improved without any change in HRV		Frequency domain: HF Time domain: RMSSD	
	Sarlon et al [50]	89 with MDD		HRV: blood volume pulse finger clip sensor Skin conductance: Velcro tape with integrated Ag/AgCl electrodes Skin temperature: Nexus temperature Beat-to-beat interval: elastic belt with a breathing sensor 3 conditions: baseline sitting for 5 minutes, with emotion-induced stressors (recall an unpleasant stressful experience), and relaxed state after 300 seconds		No association was found between HRV and symptom severity		Frequency domain: VLF, LF, HF, LF-to-HF ratio Time domain: SDNN, RMSSD, beat-to-beat interval Skin conductance Skin temperature	
**EDA studies**									
	Kim et al [51]	30 with MDD and 37 in HC		ProComp Infiniti (SA7500, computerized biofeedback system, thought technology) EDA during 5 experimental phases: baseline, mental arithmetic task, recovery from the stress task, relaxation task, recovery from the relaxation task		Classifying participants with MDD versus controls: 74% accuracy, 74% sensitivity, 71% specificity Stress and relaxation tasks were the most relevant		MSCL^r^, SDSCL^s^, SKSCL^t^, and NSSCR^u^	
	Kim et al [52]	30 with MDD and 31 in HC		As per Kim et al [51]		Classifying participants with MDD versus controls: 70% accuracy, 70% sensitivity, 71% specificity		MSCL, SDSCL, SSCL^v^, MSCR^w^, NNSCR^x^, and poststroke depression	
	Litwińska-Bołtuć et al [53]	97 with MDD Follow-up at 1 year		EDOR (Electro Dermal Orienting Reactivity) test (Emotra AB)		Hyporeactive patients: relapse or recurrence of depression was nearly 5 times higher		MSCR	
	Pedrelli et al [54]	31 with MDD Follow-up at 8 weeks		2 E4 Empatica wristbands, one on each wrist for 22 hours/day, 7 days/ week Smartphone sensor data: movisensXS phone app (movisens GmbH)		Correlations between the models’ estimate of HDRS scores and clinician-rated HDRS: from OR^y^ 0.46 (CI 0.42-0.74) to OR 0.7 (CI 0.66-0.74)		Empatica: EDA: MSCR, PSCR^z^, AASCR^aa^ Peripheral skin temperature Heart rate 3-axis accelerometer and sleep characteristics Smartphone data: mobile-based social interactions (number of calls, SMS text messages), activity patterns (walking), number of apps used	
	Smith et al [55]	11 with MDD 16 in HC		Shimmer3 GSR+ unit with Shimmer Optical Pulse Sensing Probe; at rest with eyes closed for 3 minutes		81% accuracy for detecting depression with HRV No benefit to including skin conductance response to improve accuracy		HRV Time domain: SDNN, beat-to-beat interval Nonlinear: SD1, SD2 Skin conductance response: MSCR, SDSCR^ab^, PSCR, and AASCR	

^a^HC: healthy control.

^b^MFCC: mel-frequency cepstral coefficient.

^c^BD: bipolar disorder.

^d^AUC: area under the curve.

^e^tDCS: transcranial direct current stimulation.

^f^HDRS: Hamilton Depression Rating Scale.

^g^NVB: nonverbal behavior.

^h^AU: action unit.

^i^ECG: electrocardiogram.

^j^HF: high frequency

^k^RMSSD: root-mean-square surface distance.

^l^LF: low frequency

^m^SDNN: SD of the NN (R-R) intervals.

^n^SD1: SD of points perpendicular to the major axis of the Poincaré plot.

^o^SD2: SD of points along the major axis of the Poincaré plot.

^p^PNN50: proportion of NN50 divided by the total number of NN (R-R) intervals.

^q^VLF: very-low frequency.

^r^MSCL: mean amplitude of the skin conductance level.

^s^SDSCL: standard deviations of the skin conductance level.

^t^SKSCL: skewness of the of the skin conductance level.

^u^NSSCR: nonspecific skin conductance response.

^v^SSCL: slope of the skin conductance level.

^w^MSCR: mean amplitude of the skin conductance response.

^x^NNSCR: number of nonspecific skin conductance responses.

^y^OR: odds ratio.

^z^PSCR: peak skin conductance response.

^aa^AASCR: average amplitude skin conductance response.

^ab^SDSCR: standard deviations of the skin conductance response.

### Major Depressive Disorder

#### Speech Analysis

Prosodic abnormalities in patients with depression are well known and a monotonous speech or reduced prosody can be easily and frequently heard. It is assumed that cognitive and physiological changes in depression could affect speech production, and changes in the automatic and somatic nervous system cause disturbances in muscle tension and respiratory rate. These changes will not only influence vocal folds and vocal tract dynamics, but also constrain articulatory movement [56].

Speech analysis, which consists of automatically extracting vocal and linguistic features from audio signals, offers the possibility to detect and measure these changes and serves as an additional objective assessment of depression. Traditionally, acoustic features can be divided into 5 types: temporal, prosodic, spectral, source, and filter features. Temporal features, defined as speech prosodic timing measures, appear to be a promising measure of depression and its severity [57].

Yamamoto et al [31] used 3 temporal features in a prospective follow-up study: speech rate, pause time, and response time. They found a correlation with scores on the Hamilton Rating Scale for Depression (HAM-D). Mundt et al [25] found 5 prosodic timing measures to be significantly correlated with depression severity: total speech time, total pause time, percentage pause time, speech pause ratio, and speaking rate. Xu et al [30] found that people with depression have a longer response time and interrupt their communication partner more often. To summarize, people with depression used a much larger percentage of words related to the past and sadness emotions. Similarly, Shinohara et al [27] used a “vitality” score based on emotional elements of speech (ie, * joy*, *anger*) and showed a negative correlation between the *vitality* score and HAM-D scores. Arevian et al [22] analyzed the types of word used and found, among others, more negative emotion and less complex word use in patients with depression.

Finally, Alghowinem et al [21] and Hönig et al [23] found that the average syllable duration could be positively correlated with depression severity.

With prosodic features in people with depression, reduced fundamental frequency (F0) range and average are frequently found [23,24,26,58]. F0 corresponds to the lowest frequency of the speech signal perceived as pitch. It could explain the depressive monotonous speech and could be a consequence of disturbances in laryngeal muscle tension due to psychomotor retardation. The latter may also explain reduction in F0 variability [56,57]. However, some studies also report no significant correlation between F0 variables and depression [25,28].

Several studies on depression showed a shift in spectral energy, and mel-frequency cepstral coefficients (MFCCs) are often used as spectral features. Spectral features characterize the spectrum of speech, which at a given time correspond to the frequency distribution of the speech signal. Taguchi et al [28] showed that the second dimension of MFCC allowed a significant discrimination between patients with depression and controls. Further, Wang et al [29] found that MFCC 5, MFCC 7, and loudness were consistently lower in people with depression. According to the task designed during speech recording, MFCC 3 and F0 were significantly lower than in people with depression than in healthy people.

Finally, source features such as harmonic-to-noise ratio (HNR), jitter, and shimmer also tend to increase with depression severity, supporting the hypothesis of a more breathy phonation in depressed speech [26]. This could be explained by a more open and turbulent glottis, linked to a reduction in laryngeal muscle tension. The study by Hönig et al [23] also supports this hypothesis. Alghowinem et al [21] showed that not only shimmer and jitter but also loudness and MFCC features are part of the general characteristics of depressive speech.

#### Nonverbal Behavior Analysis

Traditionally, evaluation of clinical depression is based on patients’ verbal information from psychological interviews and rating scales or self-report questionnaires. However, verbal analysis has several limitations. First, patients may underreport or overreport depressive symptoms due to different reasons (eg, social stigma). Second, it can be assumed that most human communication is taking place through nonverbal behavior. Indeed, a large part of nonverbal behavior is mostly outside of conscious control, and these cues and signals may differ from verbal reports. Facial expression, gestures, and body postures are mainly involuntary, and therefore represent a privileged way of expressing feelings and emotions [35,59]. Girard et al [38,39] showed that when the severity of depressive symptoms was high, participants made fewer affiliative facial expression such as smiling, and more nonaffiliative facial expression such as contempt. They also showed that patients exhibited diminished head motion (amplitude and velocity). These results support the social withdrawal hypothesis in depression. In fact, rather than affective valence with an increase or decrease in facial expression, it is the social-communicative value with affiliative expression that seems to be affected in depression. Thus, nonverbal behavior may serve to maintain interpersonal distance while facilitating social withdrawal.

Fiquer et al [35] supported the assumption that depression involves behaviors related to social withdrawal and negative feelings. Crying, asymmetric smiling, and motionless head/eyebrows were associated with higher levels of depression severity, while patients’ illustrative gestures, defined as “the hand and arm movements used to support the speech,” were associated with a lower level of depression severity.

Moreover, decrease of negative facial and head indicators and increase of eye contact and yes nodding accompanied clinical improvement, whereas illustrative gestures did not [35]. In another study, Fiquer et al [37] compared nonverbal behavior in patients with depression versus controls. They showed that patients with depression displayed higher levels of negative nonverbal behaviors (shrug, head and lips down, adaptative hand gesture, frown, and cry) and lower levels of positive nonverbal behaviors (eye contact and smile). The authors raise the important role of these behaviors in social withdrawal [37]. Nevertheless, the aforementioned nonverbal behaviors was not associated with depression severity before treatment, which suggests its independence.

The authors concluded that certain typically found nonverbal behaviors could represent a predisposition to depression possibly influenced by personality features. These social symptoms expressed through nonverbal behavior may be the last ones to vanish [37].

On the contrary, Fiquer et al [36] studied nonverbal variables such as speaking effort, encouragement, attunement, and change in attunement, and found no association between these behaviors and the severity of depression. Finally, several recent studies achieved classifying depression with high accuracy using video analysis with head and facial movements [34,42], only head movement [33], facial expressivity [41], or with head pose and eye activity [32]. Guo et al [40] found that the highest accuracy of depression recognition was when patients were watching film clips of positive and negative emotional stimuli compared with other tasks.

In a recent paper, Alghowinem et al [60] used feature selection methods and found that some features have a high capability for distinguishing between depression severities. For speech analysis, the strongest features were the temporal features, F0, HNR, formants, and MFCC; and for behavioral analysis, it was the left-right eye movement and gaze direction and the yaw head movement. The authors showed that these features outperformed all other features in depression detection.

The studies mentioned so far demonstrate the usefulness of behavioral analysis in understanding the mechanisms that underlie the onset and course of depression. These analyses are less influenced by conscious control compared with verbal communication [59], which could make them a reliable objective measure.

#### Physiological Measures

##### Heart Rate Variability

HRV corresponds to the beat-to-beat variations in the heart rate over a given period. HRV appears to be a good reflection of the heart’s ability to modulate its rhythm in response to external and internal stimuli. HRV is regulated by the autonomic nervous system and, as a result, can inform of its functioning. HRV is related to the influence of the parasympathetic nervous system (PNS) and the sympathetic nervous system (SNS) [61]. Three parameters are commonly used: high-frequency HRV (HF-HRV; 0.15-0.40 Hz), low frequency HRV (LF-HRV; 0.04-0.15 Hz), and the LF-to-HF ratio. HF-HRV is mainly under the control of the PNS, whereas LF-HRV is under the influence of both PNS and SNS, but mostly the SNS. The LF-to-HF ratio is considered an index of sympathovagal balance [61].

In addition, time-domain parameters such as the root-mean-square of successive differences (RMSSD) and the SD of normal-to-normal intervals (SDNN) are frequently used [62]. Normal-to-normal is the interval between 2 heartbeats. HRV can be an indicator of the ability to regulate stress and emotions, which is particularly relevant in psychiatric disorders [63]. Furthermore, a good cardiovascular adaptability can be reflected by a high HRV [64], while LF can be a risk factor of cardiovascular diseases [65]. A meta-analysis in depression [66] showed significant reductions in frequency domain parameters such as HF, LF, and very-low frequency (VLF) HRV as well as a significantly higher LF-to-HF ratio compared with healthy controls. Significant reductions in time domain parameters were further found with reduced RMSSD, SDNN, and interbeat intervals.

Some recent studies have focused on different clinical subdimensions of MDD. Lee et al [48] showed a positive correlation between fatigue-related items of the Hamilton Depression Rating Scale (HDRS) and LF-HRV. The latter was positively correlated with the items “midnight insomnia” and “slower thinking.” In the same line, Adolph et al [43] found that HF-HRV in reaction to watching a sad film was predictive of higher scores on suicidal ideation, whereas HF-HRV at rest was not predictive of suicidal ideation. Further studies investigated the relationship between HRV and treatment response. First, Hartmann et al [46] found that patients with MDD normalized their HRV after treatment, especially for HF-HRV and LF-HRV. Further, Giurgi-Oncu et al [45] showed a normalization of HRV parameters (including HF, RMSSD, and SDNN) with the depression treatment by therapy and medication. Kircanski et al [47] predicted treatment success among patients with depression with or without anxiety. The authors found that a higher HF-HRV in pretreatment had better outcomes than lower HF-HRV in anxious depression, whereas lower HF-HRV had better outcomes than higher HF-HRV in nonanxious depression.

By contrast, Sarlon et al [50] found no association between HRV and depressive symptoms severity. Neyer et al [49] showed in a prospective study with HRV measures before and after treatment that depressive symptoms improved without change in HRV, suggesting a more complex relationship.

Finally, Fernandes et al [44] explored the association between HRV and nonverbal behavior using a validated ethogram. Results showed a positive correlation among HRV (HF and RMSSD) and positive nonverbal behaviors, but no association between HRV and negative affect. The authors suggested that HRV and nonverbal behaviors could be regulated by vagal activity, which may be related to the social engagement system.

##### Electrodermal Activity

The most basic indicators of the state of the autonomic nervous system are heart rate and EDA. EDA can measure electrical conductance of the skin, which depends on the quantity of sweat secreted by glands in the hypodermis and reflects sympathetic nervous activity.

EDA has a tonic and a phasic component. The former is measured in the skin conductance level (SCL) and the latter with the skin conductance response (SCR) [67]. EDA can be used as an indicator for emotional reactivity [68]. In fact, rather than the valence of emotion, it is the intensity that seems related to EDA [69] and negative stimulation induces more extensive reactions than positive [70]. Concerning emotional reactivity, we distinguish labile and stable patients for EDA. EDA labile patients could be described as calm and deliberative, whereas EDA stable patients tend to be more irritable, emotionally expressive, and active. EDA labile patients would be dependent on anxiety traits and better control for potentially threatening stimuli [71].

Several studies show that EDA could be an indication of nonconscious emotional processes [72-74]. A recent systematic review [67] showed lower EDA, especially lower SCL and SCR, in patients with depression compared with healthy controls. Others studies report that, compared with patients with depression also experiencing agitation, those with psychomotor retardation or symptoms of inhibition have lower EDA levels [75-77]. Moreover, patients with “psychotic ” and “endogenous” depression could have lower EDA than “nonendogenous” depression [77-79].

Other recent studies showed moderate to high accuracy in classifying patients with MDD and healthy controls [51,52], such as that by Smith et al [55], but the latter used HRV and SCR for depression detection and found no benefit in including SCR to improve accuracy. In addition, Pedrelli et al [54] showed that EDA and HRV features from wearables as well as activity level and sleep parameters could provide an estimate of changes in severity of depressive symptoms.

In a cohort of patients with MDD at 1-year follow-up, Litwińska-Bołtuć et al [53] concluded that SCR hyporeactivity was associated with recurrent episodes and relapse, and recurrence of depression was almost 5 times higher than that in the reactive patients.

#### Concluding Remarks

Based on these aforementioned studies, the digital phenotype of MDD could be identified as follows: a reduction in certain speech temporal features (ie, speech rate or speech time), an increase in others (ie, pause time or response time) [25,31], and a change in the type of words used (ie, less complex and more emotionally negative) [22,27]. In addition, prosodic features (ie, F0 range and average) [23,24,26,58], variability [56,57], spectral features [28,29], and source features (ie, HNR, jitter, shimmer [21,26]) are changed.

The behavior of patients with depression could be characterized by a more negative nonverbal behavior (ie, head motion, facial expressivity, and hand or body gestures) [35,38,39]. But more than the valence, it seems that it is the affiliative behaviors that are impacted, supporting a social withdrawal [38,39]. In fact, certain patterns of behavior when interacting with others may predict the severity of symptoms or treatment response [36,59,80-83]. Finally, physiological measures are mainly lowered for both HRV (ie, HF, LF, VLF, and time domains) [66] and EDA (ie, SCR and SCL) [67].

### Bipolar Disorder

#### Speech Analysis

Several studies managed to classify the course of mood episodes or relapses with high confidence. For instance, speech pause duration [84] and number of longer pauses [85] are significantly higher in depressive states than in a hypomanic or euthymic state. Concerning prosodic and source features, Guidi et al [85-87] repeatedly found an increase of F0 in hypomanic states compared with euthymic or depressive states. Similar results were also obtained by Vanello et al [88]. Likewise, filter features seem to increase with manic mood [89], especially F1 and F2 formants, which correspond to the first and the second peak in the spectrum that results from a resonance of the vocal tract.

Finally, many studies extracted several speech features (ie, the openSMILE toolkit), achieving good classification accuracies [90,91]. Classification of depressive states could be obtained with an area under the curve (AUC) value of 0.78 and manic and mixed states with an AUC value of 0.89 [92].

#### Physiological Measures

##### Heart Rate Variability

A meta-analysis [93] found reduced HRV compared with healthy controls. In particular, LF-HRV was reduced but no differences in either HF or LF-to-HF ratio were found. However, these analyses did not consider different mood phases. Recent studies were interested in the exploration of HRV during a specific phase of BD. First, Wazen et al [94] showed that several HRV parameters (HF-HRV, time-domain, and non-linear domain measures) could increase when patients go from the mania phase to the euthymic phase. Conversely, Faurholt-Jepsen et al [93] reported an increased HRV in manic states compared with depressive and euthymic states, but no difference between the depressive and euthymic states. Hage et al [95] found that LF-HRV and heart period were significantly lower in patients with BD compared with healthy controls. However, after 8 weeks of treatment, there were no significant changes in HRV parameters.

The link between disease severity and HRV has also been explored. Benjamin et al [96] found a significant association between HF-HRV and disease severity. In the same way, Ortiz et al [97] found that longer illness duration, higher number of depressive episodes, longer duration of most severe manic/hypomanic episode, comorbid anxiety disorders, and a family history of suicide were associated with a reduced HRV. Moreover, the severity during a depressive episode was associated with lower HRV. Finally, Freyberg et al [98] compared 20 newly diagnosed BD cases, 20 unaffected first-degree relatives, and 20 healthy controls, and demonstrated that HRV did not differ in any measures between the 3 groups.

Lastly, 2 studies have tried to distinguish bipolar depression and MDD, in terms of HRV. First, Chang et al [99] studied a population of patients with depression and BD type II and found that, compared with patients with MDD, they had lower total HRV, with lower HF and LF-HRV but higher LF-to-HF ratio. The authors concluded that HRV may aid in the differential diagnosis of bipolar depression type II and unipolar depression. Second, Hage et al [100] supported these findings by comparing patients with MDD with those with BD depression including BD type I, type II, and “not otherwise specified.” Indeed, patients with MDD had significantly higher baseline sinus arrythmia (ie, HF-HRV) and LF-HRV in comparison to patients with BD.

##### Electrodermal Activity

Greco et al [101] showed that EDA levels could differentiate the phases of BD (depressive, euthymic, and mixed states). Indeed, electrodermal hypoactivity could be a rather stable trait of patients with depression and may reflect euthymia or remission when EDA increases [101]. Lemaire et al [102] measured the intensity and duration of physiological responses to affective stimuli. For this purpose, they used EDA and the heart interbeat intervals. The authors concluded that affective dysregulation, a key dimension of BD, could be measured as an increased affective reactivity to neutral pictures and reduced maintenance of subjective affective responses to all type of pictures, irrespective of the clinical state.

#### Concluding Remarks

For the digital phenotype of BD, we found that, in the depressed phase, speech changed in temporal (ie, decreased speech pause) and prosodic features (ie, decreased F0), but both increased when patients go into the hypomanic phase [84-88].

Independent of the phase, HRV appears reduced (ie, LF) [93], but the change in HRV between phases remains unclear [94,95,103]. For EDA, an electrodermal hypoactivity in a depressive state is reported, which increases when patients move toward euthymia [101].

### PTSD and Psychological Trauma

#### Speech Analysis

The link between trauma and voice has been likewise explored. Monti et al [104] reported a significant relationship between voice fundamental frequency and the “total trauma” reported with the Childhood Trauma Questionnaire. Interestingly, after trauma recall, vocal jitter and voice irregularity (roughness) were strongly related to the existence of childhood trauma [104]. This suggests that variations in vibratory patterns of the vocal folds could have a relationship with childhood trauma recall. In another study, Monti et al [105] also found an association of anxious attachment and childhood neglect with intensity/loudness measures in singer’s voice. Anxious attachment was also positively correlated with jitter and irregularity. Moreover, in speech content, trauma narrative shows an increased use of first-person singular pronouns [106,107] and changes in narrative coherence [108,109].

As in depression, voice markers including F0, jitter, shimmer, and HNR are also found in PTSD [110,111]. Speech rate in both depression and PTSD was shown to be negatively correlated with severity of symptoms. Reduced tonality in vowel space is found in self-reported symptoms of both PTSD and depression [112]. However, in PTSD without MDD, voice markers showed slower and more monotonous speech and less change in tonality [113]. Finally, a recent study showed that audio intensity and reduced pitches per frame could predict PTSD, whereas reduced frequency of positive words seemed to rather predict depression [106].

#### Nonverbal Behavior Analysis

A core symptom of PTSD is an exaggerated startle response [2]. In their study on patients with PTSD, McTeague et al [114] focused on the eye-blink startle response to narrative texts corresponding to traumatic stories. The authors found a heightened startle reflex response during trauma imagery, which was also evident in reactivity to anger, panic, or physical danger compared with controls. Surprisingly, participants with multiple traumas showed a blunted reactivity compared with participants with a single trauma. Furthermore, participants with multiple traumas had more chronic and severe PTSD with more anxiety and mood comorbidity [114].

In the same paradigm, Blechert et al [115] studied the bodily startle response to electric stimuli in individuals with trauma, with and without PTSD. The results implied a primary response characterized by reflexlike facial and torso movement and a secondary response characterized by an emotional facial response. Besides, participants with PTSD had a stronger initial startle response and a more negatively valenced secondary facial expression compared with controls with trauma. Beyond the startle response to threat, several studies with eye tracking showed that higher levels of PTSD symptoms were associated with an increased attentional bias toward threat [116]. Some authors assumed that negative affect in PTSD could have an important role in maintaining this attention bias [117].

More generally, Katz et al [118] emphasized the importance of trauma disclosure. The authors analyzed 40 video interviews of children who have suffered an abuse, but only half of them disclosed it. Children who did not disclose the abuse showed specific nonverbal cues, such as more physical disengagement, than children who disclosed. Schultebraucks et al [106] performed deep learning audio- and video-based classification of depression versus PTSD for 81 participants, 1 month after their admission to a trauma center following a traumatic exposure. The authors successfully classified between PTSD and MDD status with a high discriminatory accuracy. Likewise, several voice and video markers have been identified as predictive of either PTSD or MDD. In a video analysis, higher fear and anger expressivity were predictive of PTSD, while higher contempt expressivity was important for the classification of MDD. In an audio analysis, an increased use of first-person singular pronouns, lower audio intensity, and reduced pitch were important features for the PTSD classification, whereas a reduced frequency of positive words was predictive of depression.

#### Physiological Measures

##### Heart Rate Variability

Several studies have investigated HRV in PTSD and suggested reduced parasympathetic activity in PTSD. Indeed, 2 meta-analyses found that HRV is lower both at rest and during stress in PTSD compared with controls. Particularly, RMSSD and HF-HRV were reduced, while SDNN and LF-HRV were also reduced, but with a smaller effect. Furthermore, a higher LF-to-HF ratio was found in PTSD [119,120]. Another meta-analysis [121] investigated the relationship between early life maltreatment (ELM) and resting-state HRV. Although no evidence for a relationship was found in comparative and correlational meta-analyses, the meta-regression analysis disclosed interesting results regarding both age and presence of psychopathology. In fact, studies including older-aged samples reported greater reductions in resting-state HRV in association with more severe ELM. Moreover, in clinical samples, compared with nonclinical samples, the authors found stronger reductions in resting-state HRV in ELM-exposed individuals compared with those nonexposed. The authors assumed that ELM could lead to alterations in the development of the autonomic nervous system, especially to an insufficient increase in vagal activity during childhood and adolescence, leading particularly to deficiencies in affective regulation that could increase the risk for psychopathology [121].

Finally, Stone et al [122] compared HF-HRV in women with depression with and without a history of childhood emotional abuse (CEA) and in controls without CEA, and reported that women with depression with CEA exhibited lower HF-HRV than both groups without CEA.

##### Electrodermal Activity

In patients with PTSD, higher subjective levels of intrusive reexperiencing have been linked to physiological reactivity during trauma recall, while acute dissociative symptoms could show reduced physiological reactivity to trauma memories. Actually, increased physiological reactivity was not associated with the self-report of memory avoidance or dissociation but rather with the degree of memory intrusiveness felt [123,124]. Thus, it can be hypothesized that persistently high physiological reactivity may facilitate chronic arousal dysregulation that disrupts neural systems and could lead to maintain the intrusiveness of remembering [125,126].

Patients with PTSD showed an increased physiological reactivity during trauma recall, while patients with depression had a low physiological response to the memory of an event that they associated with the onset of their depression [126]. Although some researchers suggest a possible attenuation of physiological reactivity in patients with PTSD by depressive symptoms [114], when comparing trauma recall between PTSD with and without depression, no blunting effect was found by depressive mood on physiological reactivity [126]. D’Andrea et al [127] studied individuals exposed to trauma and showed different autonomic reactivities in response to startling sounds according to the type and symptomatology of trauma. Indeed, blunted autonomic reactivity was found in participants with significant symptoms and early exposure to multiple traumas. Conversely, more attenuated trauma exposure had large heart rate acceleration and SCRs.

#### Concluding Remarks

In PTSD, modifications in speech prosodic and source features are found as in depression [104,110-113], but specific changes are reported in the speech content, especially during trauma narrative or trauma recall, that is, use of first-person singular pronouns [105,106], and changes in narrative coherence [108,109].

In the same way, behavior analysis shows a specific startle response during trauma recall (ie, eye blink or bodily startle) [114,115]. Besides, facial expressivity with higher fear or anger could be predictive of the presence of PTSD [106].

In physiological measures, for HRV, PTSD studies indicated a reduced parasympathetic activity (ie, HF, LF, and time domains) [119,120] as in depression. However, the presence of trauma in people with depression could further decrease HRV (ie, HF) [122]. For EDA, the presence of PTSD, especially with memory intrusiveness felt, leads to an increased EDA reactivity during trauma recall while acute dissociative symptoms could have the opposite effect [123,124,126].

## Discussion

### Principal Expectations

In this review, we were interested in the state-of-the-art application of new digital measurements to assess symptoms in patients with several psychiatric conditions. In particular, we explored digital markers through speech and behavioral analysis, as well as physiological measures such as HRV and EDA. We focused our research on depression and its different clinical characteristics.

### Limitations

Although several databases have been included, relevant papers may have been missed due to the choice of keywords. This paper is not intended to be a systematic review and a broader review could explore other interesting areas. We included a wide range of study designs due to the exploratory nature of our review but the design of the included studies also limited the review. Other reviewers have attempted to reduce the study selection bias but they have acknowledged the possibility of subjectivity in their analyses of the findings.

In addition, we noticed that overall, most studies only investigated the use of 1 digital measure, except for a few that tried to combine, for instance, the measure of EDA with HRV or speech with behavioral analysis.

Apart from a few ethological analyses, most studies analyzed the patient alone, without being in interaction. However, social interaction, especially during clinical interviews, seems to be the most important source of information for clinical assessment.

Moreover, we frequently found that each study used different measures that raise a problem of harmonization. For example, in speech analysis, some studies assessed the type of word used, prosodic or spectral features, or even analyzed a “toolkit” with hundreds of features. Further, certain studies investigated features extracted from free speech, whereas others focused on more constrained speech tasks.

Video analysis can be heterogeneous as well, in that some studies are interested in eyes, head, or torso movements at rest or during an evaluation. In HRV assessment, several studies used electrocardiogram but others used a finger clip sensor. In the same way, EDA can be measured with or without a concurrent task and with different types of technological tools, with variable degrees of precision.

Finally, we did not take into consideration articles dealing with depression with comorbid anxiety. Future research on this topic would be relevant to complete our findings.

### Implications for Practice

Assuming that digital markers would help in the differential diagnosis for an MDE, clinicians will be able to make an early diagnosis. Thus, psychotherapy and drug treatment could be introduced early, and diagnostic and therapeutic mistakes could be limited.

Despite the lack of comparative studies, promising digital markers appear to emerge from this review, particularly within physiological measures such as HRV. In fact, patients with bipolar depression had more severe decrease than those with unipolar depression (ie, HF and LF-HRV) [99,100], whereas the presence of comorbid PTSD in MDD seems to induce a greater reactivity of physiological measures, especially when the clinician touched upon psychological trauma [126]. This reactivity with PTSD also seems to be reflected in speech and nonverbal behavioral analysis, which suggests other candidates for digital markers [108,109,114,115].

### Conclusion and Future Directions

Despite many limitations, the studies detailed in our review showed promising results on the usefulness of digital phenotyping for the differential diagnosis of MDE. The authors believe that additional research is needed to better understand the potential value of digital phenotyping in clinical practice. Indeed, what should be relevant is a prospective and observational study in which different digital tools will be combined during recordings of social interaction. At least three distinct groups would be required: MDD, bipolar depression, and depression comorbid with PTSD. For each group, it would be interesting to merge speech, behavior, and physiological measures extracted from recordings of clinical interaction between patients and clinicians. By designing larger and comparative studies, a distinct digital phenotype could be defined for each of the aforesaid 3 groups. Thus, digital markers to help differential diagnosis could emerge.

Moreover, if large prospective studies are conducted, validated digital markers could be used for follow-up and for predicting treatment response or risk of relapse. Digital phenotyping could therefore contribute to personalized and precision medicine.

## Appendix

Multimedia Appendix 1 Search strings.

Multimedia Appendix 2 Summary of speech analysis, nonverbal behavioral analysis, HRV, and EDA studies in individuals with BD, PTSD or psychological trauma. BD: Bipolar Disorder; EDA: electrodermal activity; HRV: heart rate variability; MDD: major depressive disorder; PTSD: Post Traumatic Stress Disorder.

## Abbreviations

AUC: area under the curve
BD: bipolar disorder
CEA: childhood emotional abuse
DSM-5: Diagnostic and Statistical Manual of Mental Disorders, Fifth Edition
EDA: electrodermal activity
ELM: early life maltreatment
F0: fundamental frequency
GAF: Global Assessment of Functioning Scale
HAM-D: Hamilton Rating Scale for Depression
HC: healthy control
HDRS: Hamilton Depression Rating Scale
HF: high frequency
HNR: harmonic-to-noise ratio
HR: heart rate
HRV: heart rate variability
ICD-11: International Classification of Diseases 11th Revision
LF: low frequency
MDD: major depressive disorder
MDE: major depressive episode
MFCC: mel-frequency cepstral coefficient
PANSS: Positive and Negative Syndrome Scale
PNN50: proportion of NN50 divided by the total number of NN (R-R) intervals
PNS: parasympathetic nervous system
PTSD: posttraumatic stress disorder
RMSSD: root-mean-square surface distance
RR: beat-to-beat interval
RSA: respiratory sinus arrhythmia
SC: skin conductance
SCL: skin conductance level
SCR: skin conductance response
SD1: SD of points perpendicular to the major axis of the Poincaré plot
SD2: SD of points along the major axis of the Poincaré plot
SDNN: SD of the NN (R-R) intervals
SNS: sympathetic nervous system
TAT: Thematic Appreciation Test
VLF: very-low frequency
