# Correction: Digital Phenotyping for Differential Diagnosis of Major Depressive Episode: Narrative Review

**DOI:** 10.2196/46831

**Published:** 2023-04-12

**Authors:** Eric Ettore, Philipp Müller, Jonas Hinze, Matthias Riemenschneider, Michel Benoit, Bruno Giordana, Danilo Postin, Rene Hurlemann, Amandine Lecomte, Michel Musiol, Hali Lindsay, Philippe Robert, Alexandra König

**Affiliations:** 1 Department of Psychiatry and Memory Clinic University Hospital of Nice Nice France; 2 Research Department Cognitive Assistants Deutsches Forschungszentrum für Künstliche Intelligenz GmbH Saarbrücken Germany; 3 Department of Psychiatry and Psychotherapy Saarland University Medical Center Hombourg Germany; 4 Department of Psychiatry Hopital Pasteur University Hospital of Nice Nice France; 5 Department of Psychiatry School of Medicine and Health Sciences Carl von Ossietzky University of Oldenburg Bad Zwischenahn Germany; 6 Research Department Sémagramme Team Institut national de recherche en informatique et en automatique Nancy France; 7 Research Department, Cognition-Behaviour-Technology Lab University Côte d’Azur Nice France; 8 Research Department Stars Team Institut national de recherche en informatique et en automatique Sophia Antipolis - Valbonne France

In “Digital Phenotyping for Differential Diagnosis of Major Depressive Episode: Narrative Review” (JMIR Ment Health 2023;10:e37225. doi: 10.2196/37225. PMID: 36689265; PMCID: PMC9903183) the authors made three additions to the authorship list.

The authorship is currently listed as:

*Eric Ettore, Philipp Müller, Jonas Hinze, Michel Benoit, Bruno Giordana, Danilo Postin, Amandine Lecomte, Hali Lindsay, Philippe Robert, Alexandra König*

And will be changed to read as follows:

*Eric Ettore, Philipp Müller, Jonas Hinze, Matthias Riemenschneider, Michel Benoit, Bruno Giordana, Danilo Postin, Rene Hurlemann, Amandine Lecomte, Michel Musiol, Hali Lindsay, Philippe Robert, Alexandra König*

The authors who have been recently added are affiliated with the following institutions, which are already correctly presented in the affiliation list:

Matthias Riemenschneider^3^:

*Department of Psychiatry and Psychotherapy, Saarland University Medical Center, Hombourg, Germany.*

Rene Hurlemann^5^:

*Department of Psychiatry, School of Medicine and Health Sciences, Carl von Ossietzky University of Oldenburg, Bad Zwischenahn, Germany.*

Michel Musiol^6^:

*Research Department Sémagramme Team, Institut national de recherche en informatique et en automatique, Nancy, France.*

The correction will appear in the online version of the paper on the JMIR Publications website on April 12, 2023, together with the publication of this correction notice. Because this was made after submission to PubMed, PubMed Central, and other full-text repositories, the corrected article has also been resubmitted to those repositories.

