# Supplementary material for: Digital Phenotyping for Differential Diagnosis of Major Depressive Episode: Narrative Review
Source: JMIR Ment Health. 2023 Jan 23;10:e37225. doi: 10.2196/37225 (PMC9903183; doi:10.2196/37225)
Supplement: Multimedia Appendix 1 [file mental_v10i1e37225_app1.docx]

Appendix 1 : Search String

In PubMed/MEDLINE, Web of Science, and Google Scholar databases, search terms included: [Vocal biomarkers] OR [Acoustic features] OR [Speech analysis] OR [Voice analysis] OR [Non-verbal markers] OR [Video analysis] OR [Facial expression] OR [Non-verbal behaviour] OR [Video biomarkers] OR [Biomarkers movement] OR [Ethology] OR [Heart Rate Variability] OR [Electrodermal activity] OR [Skin conductance], with each of the following terms (AND): [Depression] OR [Bipolar depression], [Bipolar disorder] OR [Mood disorder] OR [Unipolar depression] OR [Post-traumatic Stress disorder (PTSD)] OR [Posttraumatic] OR [Psychological trauma] OR [Trauma] OR [Childhood trauma] OR [Childhood abuse] OR [Childhood neglect] OR [Childhood maltreatment] OR [Early Life maltreatment] OR [Early life adversity].

A sample search query used in the PubMed database is: [(“Speech analysis” OR “Video analysis” OR “Heart Rate Variability” OR “Electrodermal Activity”) AND (“Depression” OR “Bipolar depression” OR “Mood disorders” OR “PTSD” OR “psychological trauma”)].
