# Supplementary material for: Digital Phenotyping for Differential Diagnosis of Major Depressive Episode: Narrative Review
Source: JMIR Ment Health. 2023 Jan 23;10:e37225. doi: 10.2196/37225 (PMC9903183; doi:10.2196/37225)
Supplement: Multimedia Appendix 2 [file mental_v10i1e37225_app2.docx]

**Multimedia Appendix 2**

**Table S1.** Summary of speech analysis, nonverbal behavioral analysis, heart rate variability, and electrodermal activity studies in patients with BD^a^.

| Study type and reference | | Participants | Recording setting | Principal findings | Principal features |  |  |
| --- | --- | --- | --- | --- | --- | --- | --- |
| **Speech analysis studies** | | |  |  |  |  |  |
|  | Faurholt-Jepsen et al [92] | 28 with BD | Free speech with phone call | - Sensitive and specific in the classification of manic and mixed states with AUC^b^=0.89 - AUC=0.78 for depressive state detection | - “openSMILE toolkit” |  |  |
|  | Gideon et al [90] | 37 with BD type I, II, or rapid cycling | Free speech with phone call | - AUC=mean 0.75 (SD 0.14) for mania detection - AUC=mean 0.72 (SD 0.20) for depression detection | - —^c^ |  |  |
|  | Guidi et al [86] | 9 with BD | Neutral reading task | - F0^d^ euthymic>F0 depression | - Fundamental frequency in long-term average spectrum |  |  |
|  | Guidi et al [87] | 11 with BD and 18 HCs^e^ | Comment on a series of images and neutral reading task | - F0 hypomania>F0 euthymia (neutral reading task) - LpJ (jitter) euthymia>LpJ (jitter) depression | - Average and SD of F0 and jitter |  |  |
|  | Guidi et al [85] | 11 with BD and 18 HCs | Neutral reading task | - F0median euthymic>F0median depression - F0peak euthymic>F0peak hypomania - Compared with euthymia: - ⇩ Duration pauses in hypomania states - ⇧ Depressed states | - Fundamental frequency and prosodic temporal features |  |  |
|  | Karam et al [91] | 6 with BD | Free speech with phone call | - AUC=mean 0.81(SD 0.17) for hypomania detection - AUC=mean 0.67 (SD 0.18) for depression detection | - 23 low-level features extracted (openSMILE toolkit) |  |  |
|  | Maxhuni et al [84] | 5 with BD | Free speech with phone call | - High confidence classification (85%) of the course of mood episodes or relapse | - Prosodic, temporal, emotional, and spectral features |  |  |
|  | Vanello et al [88] | 6 with BD in different states: depressed, euthymic, and hypomanic | Comment on a series of Thematic Appreciation Test images and neutral text reading | - Pitch hypomanic state>pitch euthymic>pitch depression | - Prosodic and source features |  |  |
|  | Zhang et al [89] | 30 with BD (mania state) and 30 HCs | Free speech with phone call | - ⇧ F1, F2 in patients with mania compared with HCs | - Filter and spectral features |  |  |
| **Heart rate variability studies** | | | | | | |  |
|  | Benjamin et al [96] | - 52 with BD - 52 with schizophrenia - 149 HCs | - Electrocardiogram after “vanilla task” | - In both clinical groups compared with HCs: - ⇩ HF^f^ - Statistically significant association with heart rate variability and disease severity (PANSS^g^ and GAF^h^) | - Frequency domain: HF | |  |
|  | Chang et al [99] | - 116 with BD type II in depression state - 591 with MDD^i^ - 421 HCs | - Electrocardiogram at rest | - BD depression compared with MDD: - ⇩ Mean RR^j^ intervals - ⇩ Total heart rate variability - ⇩ LF^k^ and HF - ⇧ LF-to-HF ratio | - Frequency domain: VLF^l^, LF, HF, and LF-to-HF ratio - Time domain: RR | |  |
|  | Freyberg et al [98] | - 20 with BD - 20 unaffected first-degree relatives (siblings or children) - 20 HCs | - Actiheart (Cambridge Neurotechnology) | - Heart rate variability did not differ in any measures between the 3 groups | - Frequency domain: VLF, LF, HF, and LF-to-HF ratio - Time domain: RMSSD^m^ - Activity energy expenditure | |  |
|  | Hage et al [95] | - 35 with BD in depressive state and 36 HCs - Patients were randomized to the escitalopram-celecoxib arm or to the escitalopram-placebo arm for 8 weeks in a double-blind study | - Heart rate variability by SphygmoCor - Electrocardiogram | - At baseline, BD depression was compared with controls: - ⇩ LF - ⇩ Heart period (faster heart rate) - No significant changes in heart rate variability parameters were detected over the course of the study with either treatment | - Frequency domain: LF, HF, and RSA^n^ | |  |
|  | Hage et al [100] | - 64 with MDD - 37 with BD in depressive state | Electrocardiogram at rest | - BD depression compared with MDD: - ⇩ RSA - ⇩ LF | - Frequency domain: LF and RSA | |  |
|  | Ortiz et al [97] | 53 BD (types I and II) | - 24-hour “BioModule” (Zephyr Technology), including electrocardiogram, breathing frequency, skin temperature, posture, and accelerometer measurements | - ⇩ Heart rate variability was associated with: - Longer illness duration, higher number of depressive episodes, longer duration of most severe manic/hypomanic episode, comorbid anxiety disorders, family history of suicide, and bipolar depression severity in the participants experiencing a depressive episode | - Time domain: SDNN^o^, RMSSD, SD of the average R-R intervals, and R-R triangular index | |  |
|  | Wazen et al [94] | 19 BD (manic state) | - Heart rate variability measured at rest using a Polar RS 800 CX - Measured during hospitalization in the manic state and in euthymia at discharge | - In euthymia compared with the manic state: - ⇧ HF - ⇧ RMSSD and PNN50^p^ - ⇩ LF-to-HF ratio - ⇧ SD1^q^ and SD1-to-SD2^r^ ratio | - Frequency domain: LF, HF, and LF-to-HF ratio - Time domain: SDNN, RMSSD, PNN50, and RR - Nonlinear: SD1, SD2, detrended fluctuation analysis, and entropy | |  |
| **Electrodermal activity studies** | | | | | | |  |
|  | Greco et al [101] | - 10 with BD (all phases) - 10 HCs | - Biopac MP 150 (1000 Hz) - 4 successive phases: - 5 minutes eyes closed and 5 minutes eyes open - A 6-minute slideshow of the International Affective Picture System with negative valence and 4 minutes of pictures from TAT^s^ | - Link between changes in electrodermal activity and mood states - ⇩ in depressive state | - SCL^t^: mean, maximum, and SD - SCR^u^: mean, maximum, and SD | | |
|  | Lemaire et al [102] | - 45 with BD (all phases) - 101 HCs | - Electrocardiogram and skin conductance with an SC5 digital amplifier - Positive, negative, and neutral pictures from the International Affective Picture System | - For BD: - ⇧ Affective reactivity to neutral pictures - ⇩ Maintenance of subjective affective responses to all pictures | - Maximum amplitude of SCR - Interbeat interval | | |

^a^BD: bipolar disorder.

^b^AUC: area under the curve.

^c^Not available.

^d^F0: fundamental frequency.

^e^HC: healthy control.

^f^HF: high frequency.

^g^PANSS: Positive and Negative Syndrome Scale.

^h^GAF: Global Assessment of Functioning Scale.

^i^MDD: major depressive disorder.

^j^RR: beat-to-beat interval.

^k^LF: low frequency.

^l^VLF: very-low frequency.

^m^RMSSD: root-mean-square surface distance.

^n^RSA: respiratory sinus arrhythmia.

^o^SDNN: SD of the NN (R-R) intervals.

^p^PNN50: proportion of NN50 divided by the total number of NN (R-R) intervals.

^q^SD1: SD of points perpendicular to the major axis of the Poincaré plot.

^r^SD2: SD of points along the major axis of the Poincaré plot.

^s^TAT: Thematic Appreciation Test.

^t^SCL: skin conductance level.

^u^SCR: skin conductance response.

**Table S2.** Summary of speech analysis, nonverbal behavioral analysis, heart rate variability, and electrodermal activity studies in patients with PTSD^a^ or psychological trauma.

| Study type and reference | | Participants | Recording setting | Principal findings | Principal features |
| --- | --- | --- | --- | --- | --- |
| **Speech analysis studies** | | | | | |
|  | He et al [108] | 300 trauma survivors (8 types), 150 diagnosed with PTSD and 150 without PTSD | Textual writing in response to an open-ended question and a demographic questionnaire | - Classification accuracy up to 82% and AUC^b^=0.94 - Single trauma: ⇧ words associated with PTSD symptoms - Multiple trauma: ⇧ words related to event type | - Written words |
|  | Kleim et al [107] | 163 patients with trauma | Narration of trauma event and a negative nontraumatic control event from around the same time of the assault | - Greater PTSD symptoms at 6 months could be predicted by: - ⇩ Cognitive processing words - ⇧ Death-related words - ⇧ First-person singular pronouns | —^c^ |
|  | Marmar et al [113] | 129 warzone-exposed veterans, 52 with PTSD and 77 controls | During the “CAPS” interview | - For PTSD: - The overall correct classification rate was 89.1% - ⇧ Monotonous and slow speech - ⇩ Change in tonality and activation | - Prosodic, temporal, articulatory, and spectral features |
|  | Monti et al [105] | - Study 1: 25 singers - Study 2: 26 singers | Phonetogram | - Anxious attachment, shame, and emotional neglect negatively correlated with maximum intensity in the phonetogram | - Jitter, shimmer, and harmonic-to-noise ratio to measure roughness/irregularity and breathiness/noise |
|  | Monti et al 2021 [104] | - 48 participants: 12 without a history of trauma and 36 with reported childhood trauma | - Sustained phonation - Free speech before and after trauma recall | - After trauma recall, positive relation between: - childhood trauma and its minimization with jitter and irregularity - childhood trauma with shimmer | - Fundamental frequency, noise (akin to breathiness), jitter, shimmer, irregularity (roughness), and harmonic-to-noise ratio |
|  | Scherer et al [110] | 10 scored positive for depression | Free speech | - Performance for PTSD classification: 72.09% - Performance for depression classification: 75.00% | - Glottal source signal (NAQ, QOQ, and OQ) and peak slope feature |
|  | Scherer et al [112] | 47 with MDD^d^ and 88 with PTSD | Free speech | - ⇩ Vowel space in patients that scored positively on PTSD and MDD questionnaires | - Fundamental frequency, filter features, and vowel space analysis |
|  | Xu et al [111] | 5 US soldiers with PTSD and 5 without | Free speech about their PTSD | - Average detection accuracy up to 95.88% | - Fundamental frequency, pauses, and spectral features |
| **Nonverbal behavioral analysis studies** | | | | | |
|  | Blechert et al [115] | - 60 individuals with trauma (36 with PTSD and 24 HCs^e^) - Different types of trauma: accidents, physical or sexual violence, natural disasters, war related, among others | - Video recording during and after an electric shock | - Facial and bodily startle response - Valence coding using a modified version of the Facial Expression Coding System | - In patients with PTSD compared with controls exposed to trauma: - ⇧ Initial bodily startle response - ⇧ Negatively valenced facial expression - ⇧ Anger expression - No differences were found on pain and embarrassment |
|  | Katz et al [118] | 40 children alleged of child abuse; 20 disclosed abuse, whereas the remaining 20 did not | DVD recording of interviews on children (head and legs visible) | - Stress: twitching, fidgeting, pulling hair, tapping, shifting position, licking, rigidity, self-soothing movements - Physical disengagement: shrinking, closing off, looking away, covering, getting up, turning away - Facial negative emotion: anger, fear, sadness, shame, disgust - Facial positive emotion: smiling, happiness | - Children who nondisclosed compared with those who disclosed: - ⇧ Physical disengagement - ⇧ Nonverbal cues of stress - ⇧ Physical disengagement - ⇩ Positive emotion |
|  | McTeague et al [114] | - 49 patients with PTSD and 76 HCs - Single trauma (n=22) - Multiple trauma (n=27) | - Several narrative auditory scripts were imagined by participants during listening (trauma imagery: “personal” threat for PTSD and “worst fear” for HCs, as well as other scripts with “anger,” “panic attack,” “neutral event,” among others) | - Eye blink startle response (electromyogram) - Heart rate variability (electrocardiogram) - Skin conductance level - Facial expressivity | - In patients with PTSD compared with HCs (during trauma imagery, in reactivity to anger, panic, or physical danger): - ⇧ Startle reflex - ⇧ Eye blink - ⇧ Autonomic responding - ⇧ Facial expressivity - The multiple trauma group, in comparison with the single trauma group, showed blunted reactivity associated with more chronic and severe PTSD, anxiety, and mood comorbidity |
|  | Mekawi et al [117] | 92 PTSD | Eye tracking during the “dot probe task” with photographs | - Eye tracking | - With higher levels of PTSD symptoms: - ⇧ Attention toward threat |
|  | Schultebraucks et al [106] | - 81 persons admitted in a hospital for trauma exposure - PTSD status was evaluated using the Posttraumatic Checklist for DSM-5^f^ and depression severity using the Center for Epidemiologic Studies of Depression Scale | - 1 month following hospital discharge: audio and video recording during semidirected interview with 5 questions | - Type of words used - Expressivity | - For depression classification: - ⇧ Contempt expressivity - ⇩ Positive words - For PTSD classification: - ⇧ Fear and anger expressivity - ⇧ Use of first-person singular pronouns - ⇩ Audio intensity and pitches per frame - With both audio- and video-based markers: - Accuracy to classify PTSD status: AUC=0.9 - Accuracy to classify MDD status: AUC=0.86 |
| **Heart rate variability studies** | | | | | |
|  | Stone et al [122] | - 11 women with MDD with a history of CEA^g^ - 19 MDD women without CEA - 22 HCs without CEA | - Clinical interviews followed by electrocardiogram | - Women with MDD with CEA compared with both groups without CEA: - ⇩ HF^h^ | - Frequency domain: HF |
| **Electrodermal activity studies** | | | | | |
|  | D’Andrea et al [127] | - 54 individuals exposed to trauma | - Photoplethysmograph and skin conductance coupler - Startle stimuli sounds with a “startle reflex system” | - Early exposure to multiple types of trauma: - ⇩ HR^i^ acceleration and SC^j^ - More attenuated trauma exposure and subclinical symptoms: - ⇧ HR acceleration and SC | - HR - SCR^k^ SD |
|  | O’Kearney et al [126] | 24 with PTSD, 24 with MDD, and 24 HCs | - BIOPAC MP 150 - Measures during script-driven recall about trauma and other events: “2 negative personal experiences”: the target and an “other” | - In PTSD during trauma recall compared with other events recall: - ⇧ HR acceleration and SC - In PTSD compared with MDD and HC: - ⇧ HR acceleration and SC - Higher avoidance symptoms are associated with: - ⇧ HR reactivity to trauma memory in PTSD - ⇩ HR reactivity to event memory in MDD | - HR - SC SD |

^a^PTSD: posttraumatic stress disorder.

^b^AUC: area under the curve.

^c^Not available.

^d^MDD: major depressive disorder.

^e^HC: healthy control.

^f^DSM-5: Diagnostic and Statistical Manual of Mental Disorders, Fifth Edition.

^g^CEA: childhood emotional abuse.

^h^HF: high frequency.

^i^HR: heart rate.

^j^SC: skin conductance.

^k^SCR: skin conductance response.
